# MicroRNA-212 negatively regulates starvation induced autophagy in prostate cancer cells by inhibiting SIRT1 and is a modulator of angiogenesis and cellular senescence

**DOI:** 10.18632/oncotarget.5920

**Published:** 2015-09-30

**Authors:** Malathi Ramalinga, Arpita Roy, Anvesha Srivastava, Asmita Bhattarai, Varsha Harish, Simeng Suy, Sean Collins, Deepak Kumar

**Affiliations:** ^1^ Cancer Research Laboratory, Division of Science and Mathematics, University of the District of Columbia, Washington, DC, USA; ^2^ Groton School, Groton, MA, USA; ^3^ Lombardi Comprehensive Cancer Center, Georgetown University, Washington, DC, USA

**Keywords:** prostate cancer, microRNA, miR-212, SIRT1, autophagy

## Abstract

Among a number of non-coding RNAs, role of microRNAs (miRNAs) in cancer cell proliferation, cancer initiation, development and metastasis have been extensively studied and miRNA based therapeutic approaches are being pursued. Prostate cancer (PCa) is a major health concern and several deregulated miRNAs have been described in PCa. miR-212 is differentially modulated in multiple cancers however its function remains elusive. In this study, we found that miR-212 is downregulated in PCa tissues when compared with benign adjacent regions (*n* = 40). Also, we observed reduced levels of circulatory miR-212 in serum from PCa patients (*n* = 40) when compared with healthy controls (*n* = 32). Elucidating the functional role of miR-212, we demonstrate that miR-212 negatively modulates starvation induced autophagy in PCa cells by targeting sirtuin 1 (SIRT1). Overexpression of miR-212 also leads to inhibition of angiogenesis and cellular senescence. In conclusion, our study indicates a functional role of miR-212 in PCa and suggests the development of miR-212 based therapies.

## INTRODUCTION

Prostate cancer (PCa) claimed ∼29,480 deaths in US in 2014 and estimates 220,800 new cases in 2015 [1, 2]. Clinical indications vary from indolent (requiring no treatment) to aggressive castrate resistant prostate cancer (CRPC) and metastasis, a disease more frequent in AA men. Lack of effective biomarkers to differentiate between indolent and aggressive disease and therapeutic strategies has driven the quest to identify clinically robust biomarkers to identify men with low-risk biopsy pathology with potentially aggressive tumors and new therapeutic targets to develop therapies for aggressive and resistant disease. Mechanisms of initiation and progression of prostate cancer involves poorly understood distinct molecular genetic events that disturb cell equilibrium of proliferation, cell cycle, apoptosis, autophagy and senescence included in the cancer hallmarks [3]. Sirtuin (SIRT) are highly conserved family of lysine modifying nicotinamide adenine dinucleotide (NAD^+^)-dependent class III histone deacetylases. SIRT removes the acetyl group from the ɛ-amino group of lysine residues in histones and non-histone proteins thus regulating the signaling pathways by targeting expression of genes involved. SIRT1 regulates p300/calcium binding protein, Androgen Receptor (AR) and cell cycle proteins including Rb among several other substrates modulating vital cell physiological processes (proliferation, differentiation, survival, metabolism, energy homeostasis, and aging) and pathological conditions (metabolic disease, neurodegeneration, cardiovascular disease and cancer) [4, 5]. SIRT1 expression has been reported to be higher in variety of human cancer cell lines and tissues including PCa.

Angiogenesis, the formation of new blood vessels from pre-existing vessels, is a vital requirement for the growth and metastasis of tumors. It constitutes a pivotal step in cancer progression. For the tumors to grow and have the propensity to metastasize they have to undergo the “angiogenic switch”. This switch depends on the dynamic balance between the pro- and anti-angiogenic factors. Earlier studies suggest that loss of SIRT1 resulted in down-regulation of genes involved in blood vessel development and vascular remodeling leading to significant reduction of sprouting angiogenesis and branching morphogenesis of endothelial cells. Several reports indicate that SIRT1 deacetylates and thus represses the anti-angiogenic activity of an essential negative regulator of blood vessel development, the forkhead transcription factor FOXO1 [6]. Macroautophagy (referred as autophagy hereafter) is an evolutionarily conserved self-degradative process that plays a housekeeping role by eliminating damaged organelles and misfolded or aggregated proteins *via* the lysosomal degradation pathway. Autophagy is necessary for normal cell homeostasis and its deregulation has been reported in several pathological processes including several cancers. Autophagy can be both tumor inhibiting when prolonged in response to stress of anti-cancer therapy or tumor promoting as a cell survival strategy in response to stress [7]. Autophagy can also affect chemotherapeutic and immunotherapeutic response in cancer cells making it an attractive target for development of anti-cancer drugs [8–10]. Multiple evidence including the genome-wide expression profiling of the prostates of SIRT1-/- mice and their controls identified that SIRT1 promotes autophagy [11]. SIRT1 forms a molecular complex with the genes related to autophagy and autophagosome formation, Atg5, Atg7, and Atg8. Loss of SIRT1 activity results in the acetylation of these essential components of the autophagy machinery thus leading to defects in the process [12].

MicroRNAs are highly stable noncoding small ∼22nt gene-regulatory RNAs that act primarily by targeting 3′UTRs (occasionally in 5′UTR and CDS); their roles have been studied in cancer cell survival, proliferation, and metastasis as well as biomarkers of resistance and aggressive PCa [13–17]. We recently identified differentially expressed miRNAs in PCa tissues and body fluids (serum and urine) as potential biomarkers [15, 18]. miRNA deregulation has been linked to cancer initiation and progression where miRNAs act as tumor suppressors or oncogenes, regulating multiple pathways including cell proliferation, differentiation, apoptosis, metastasis, autophagy, angiogenesis and senescence [14, 19, 20]. Because of their small size and secondary structure, mature miRNAs are highly stable for their utility as biomarkers of prediction, diagnosis/prognosis and disease progression (including survival and recurrence). miR-212 is located in tandem with miR-132 on chromosome 17p13.3 with both tumor-promoting and tumor-suppressor functions in gastric, oral and pancreatic carcinomas [21–24]. miR-212 and miR-132 belong to same family and have been reported to be generated from a stable intron of a non-protein coding gene expressed in primary neuronal cultures [25, 26]. In PCa, loss of miR-212 has been reported when compared with normal epithelium and/or stroma [17]. Multiple targets for miR-212 have been suggested and studies in multiple cancers including Lin28B in PCa [27]. Among other targets for miR-212/132, Retinoblastoma tumor suppressor gene, SMAD2, FOXA1 and SGK3 have been suggested [23, 28–30]. Although miR-212 has been studied more extensively in other cancers, its mechanistic role in PCa is not known. In the present study, we characterized the role of miR-212-3p (stated as miR-212) in modulating SIRT1 expression in PCa and studied its expression in serum and from PCa patients and PCa tissues. Given the importance of SIRT1 in modulating autophagy and angiogenesis, we also, sought to determine if miR-212 expression plays a role in controlling the autophagy and angiogenic potential of SIRT-1. Further, due to established roles of SIRT1 in influencing life span for calorie restriction and senescence in tumor cell growth, we determined the effects of miR-212 in modulating cellular senescence [31]. Our data demonstrates that miR-212 inhibits autophagy and angiogenesis by targeting SIRT1. Further we show that miR-212 induces cellular senescence. Together the study supports the role of miR-212 in the development of PCa.

## RESULTS

### miR-212 inhibits the expression of SIRT1 in prostate cancer cells

**Figure 1:**
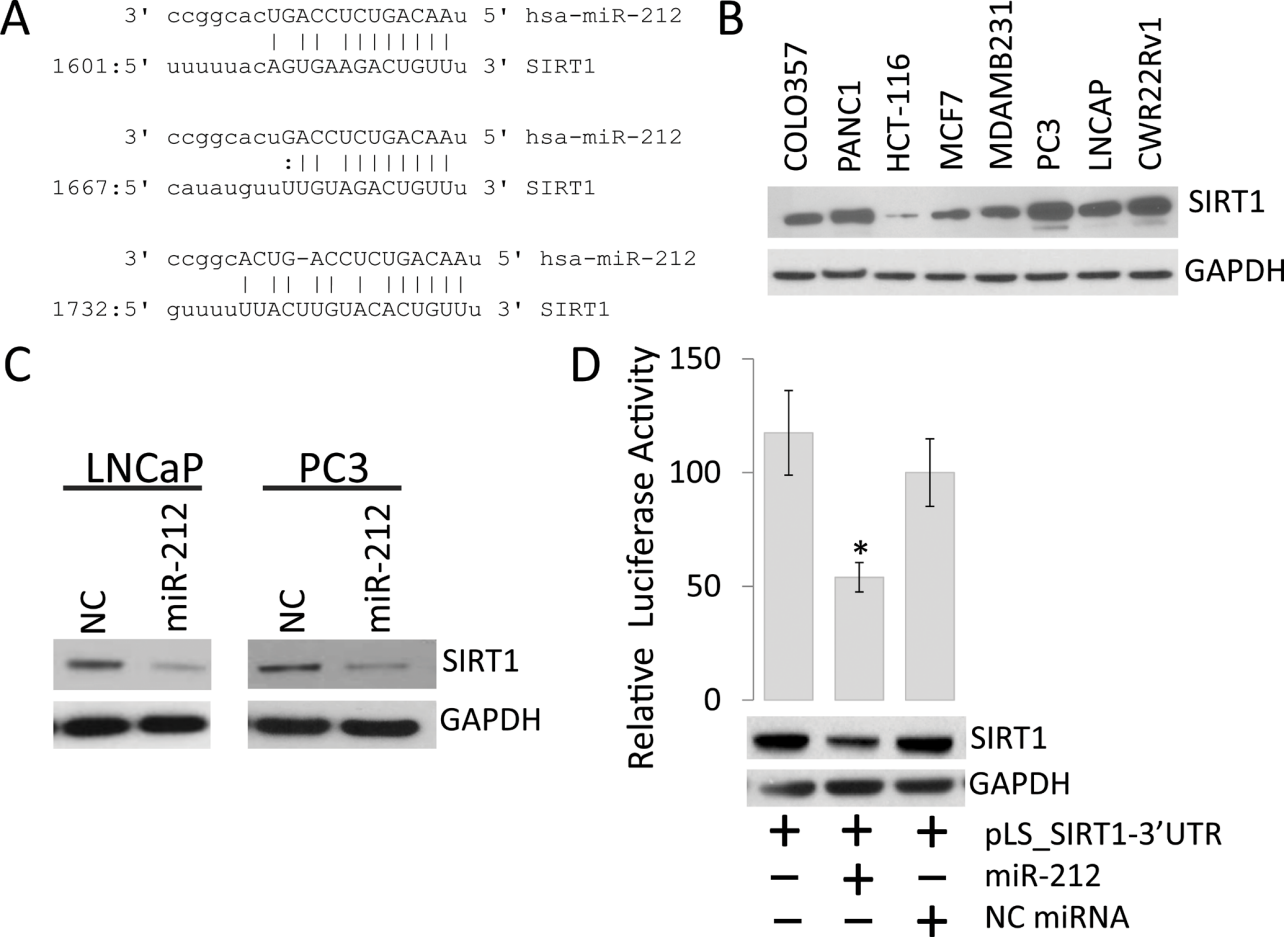
miR-212 inhibits the expression of SIRT1 in prostate cancer Cells **A.** Schematic representation of the predicted miR-212 binding site in the 3′-UTR of human SIRT1 mRNA. The predicted interaction shows the base pair matches at three regions. **B.** Endogenous expression of SIRT1 in multiple prostate, breast and colon cancer cell lines. **C.** Western blot for SIRT1 expression in LNCaP and PC3 prostate cancer cell lines after miR-212 or NC miR transfections. **D.** Luciferase reporter activity for the 3′-UTR of SIRT1 cloned in pLightswitch_3UTR vector and transfected in HEK-293 cells along with NC miR and miR-212. Luciferase activity was analyzed 48 hours after transfection to evaluate the inhibitory effect of miR-212 (*p < 0.005). Total cell lysate from the transfections was immunoblotted with SIRT1 antibody. GAPDH was used as loading control.

Multiple studies have suggested potential tumor suppressor role for miR-212 in various cancers including PCa [17, 27, 28, 32]. To understand the mechanism/s that miR-212 intervenes for PCa progression, the seed sequence of miR-212 was examined using different algorithms, TargetScan and microRNA.org. Our results show that miR-212 has 3 predicted binding sites on the 3′UTR of human SIRT1 mRNA (Figure 1A). To understand the importance of our result, we sought to determine the endogenous levels of SIRT1 in multiple cancer cell lines, including prostate cancer. Our data demonstrates the elevated expression levels of SIRT1 in all the cancer cell lines studied (Figure 1B) with plausibility that lower levels of miR-212 in PCa may result in higher levels of SIRT1. To test if miR-212 expression lowers the levels of expression of SIRT1, PCa cells were transfected with miR-212 or scrambled negative control (NC) mimics. The findings suggest a significant inhibition of SIRT1 levels upon miR-212 expression (Figure 1C). To assess whether miRNA-212 targets SIRT1, we co-transfected miR-212 mimic with luciferase reporter vector with 3′untranslated region (UTR) of SIRT1, which demonstrated that miR-212 targets the 3′UTR of SIRT1. Taken together these results implicate a role for the control of SIRT1 expression by miR-212 (Figure 1D).

### Autophagy modulation by miR-212

**Figure 2:**
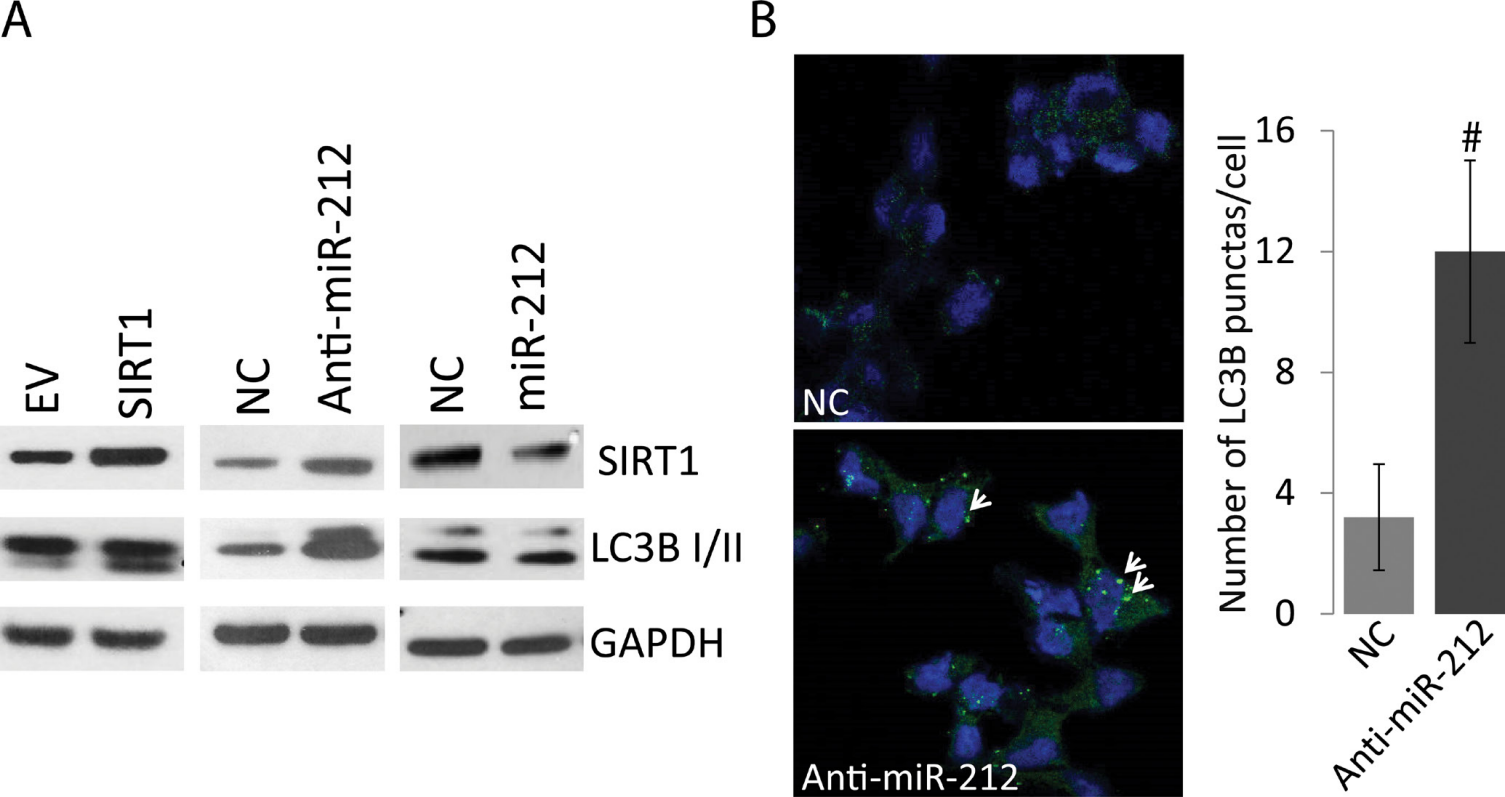
Autophagy modulation by miR-212 **A.** LNCaP cells were transfected with NC miR, miR-212 mimic and anti-miR-212 (inhibitor). Empty vector and SIRT1 expression vector were used as controls. After 48hrs of transfection, 20ug of total protein lysate was used for immunoblot analysis. Antibodies against SIRT1, GAPDH and autophagy marker, LC3B were used for analysis. **B.** Inhibition of miR-212 induces autophagy. LNCaP cells were transfected with NC and miR-212 inhibitor as described and LC3B punctas are stained by immunocytochemistry as described using LC3B antibodies. LC3B positive punctas (green; shown by arrow heads) were counted at 400x magnification on Zeiss 700 confocal microscope. DAPI was used as nuclear stain (#*p* < 0.0001).

SIRT1 levels have been associated with autophagy induction [11, 12]. Autophagy plays important but dual context dependent roles in cancer progression by promoting survival and drug resistance as well as could be apoptotic inducing. Since, miR-212 inhibits SIRT1; we asked whether miR-212 could potentially modulate autophagy in PCa cells. To determine if miR-212-induced repression of SIRT1 levels could modulate autophagy, we studied the levels of autophagy marker, LC3B-II conversion. Immunoblot analysis was performed on whole cell protein lysate after transfection of LNCaP PCa cells with negative miR, miR-212 mimic and anti-miR-212 (inhibitor). SIRT1 expression vector was used as a positive control. Our results demonstrate that expression of SIRT1 does induce autophagy as shown by LC3B conversion (Figure 2A). Further, inhibition of miR-212 using anti-miR-212 resulted in SIRT1 overexpression and induced autophagy as compared with NC (Figure 2A, 2B). Expression of miR-212 expression modestly repressed the LC3B II levels as compared to the NC and miR-212 inhibitor. Together the results indicate the role of miR-212 in modulating autophagy potentially by inhibiting SIRT1 (Figure 2).

### miR-212 inhibits autophagy induced by serum starvation

**Figure 3:**
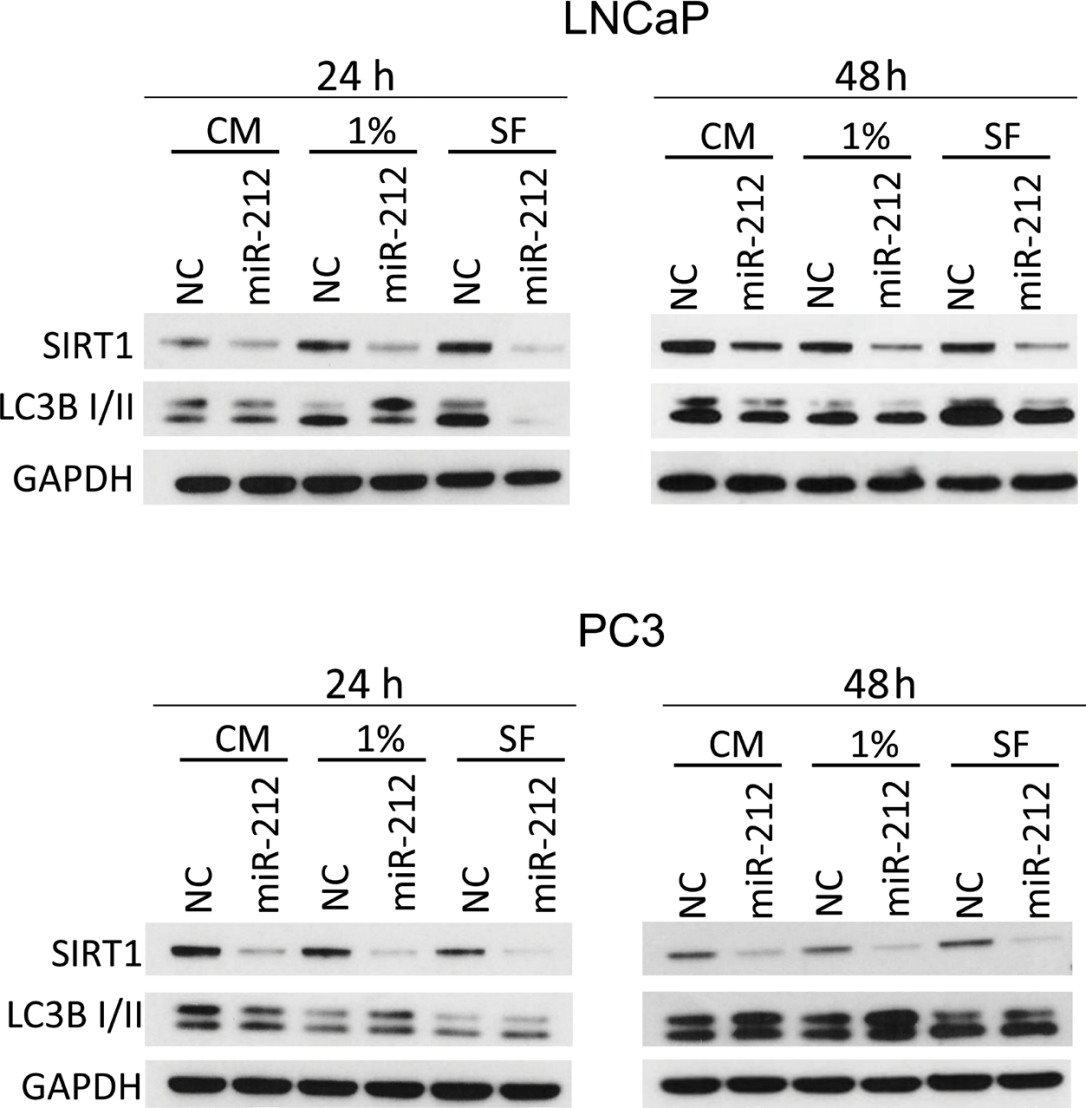
miR-212 inhibits autophagy induced by serum starvation LNCaP (top) and PC3 (bottom) cells were transfected with NC, miR-212 and cultured in complete serum media, 1% serum media and serum free media for 24 and 48 hrs. The levels of SIRT1 and LCB-I/II were determined by immunoblotting. GAPDH was used as the loading control.

Serum starvation is well recognized to induce autophagy. We asked, if miR-212 could inhibit autophagy induced by serum starvation. Androgen independent and dependent prostate cancer cell lines, PC3 and LNCaP, respectively, were transfected with NC miR and miR-212. After 24 hrs, the cells were cultured in complete, 1% serum and (autophagy inducing) serum free media (SFM). Immunoblotting with SIRT1, LC3B-I/II and GAPDH as a loading control was performed on whole cell protein lysates. Results showed the downregulation of SIRT1 expression in both cell lines after miR-212 overexpression. However, inhibition of LC3B-II expression by miR-212 was more significant in SFM than complete and 1% Serum in LNCaP cells as compared PC3 cells (Figure 3).

### miR-212 inhibits SIRT1 induced autophagy

**Figure 4:**
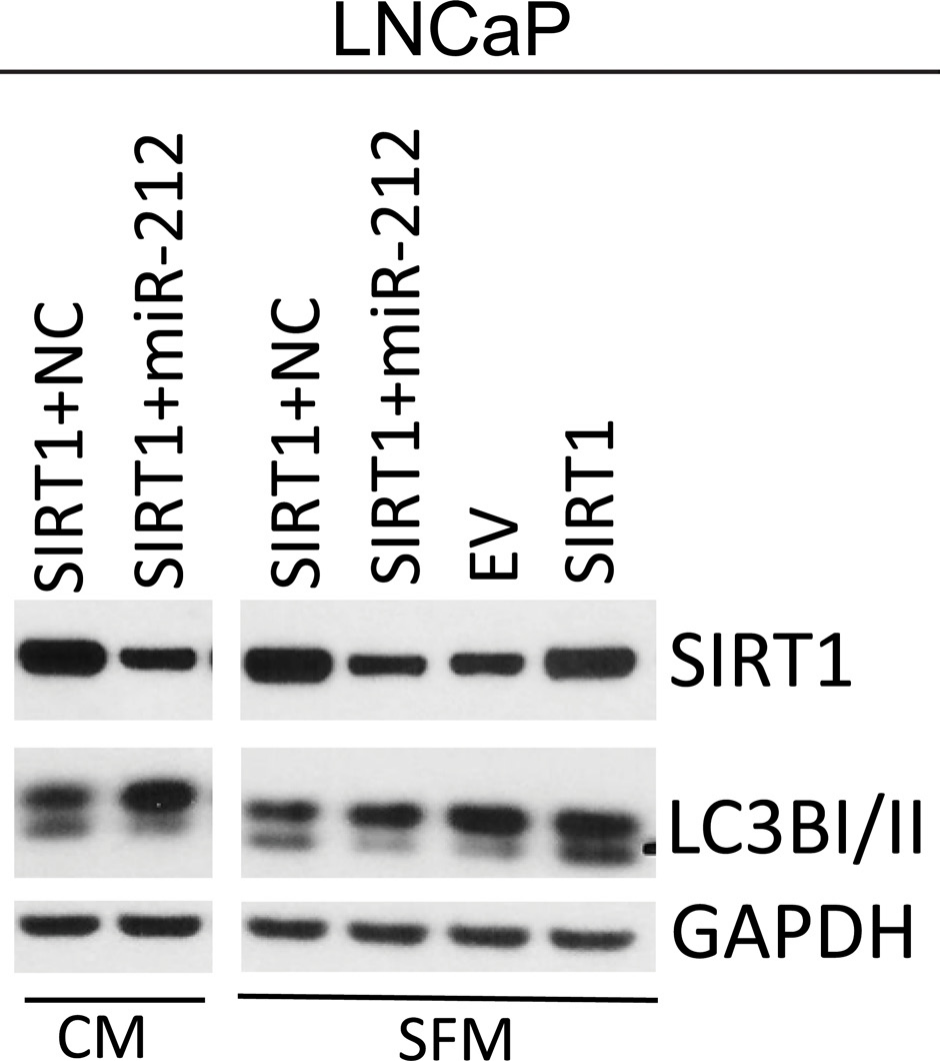
miR-212 inhibits SIRT1 induced autophagy AR-dependent LNCaP prostate cancer cells were transfected with empty vector, or SIRT1 and co-transfected with NC and miR-212 mimic. After 24h post transfection, the cells were cultured in complete media and serum free media for additional 24 hrs. SIRT1, LC3B-I/II levels were analyzed by immunoblotting. GAPDH was used as the loading control.

SIRT1 is known to induce autophagy. Since miR-212 targets SIRT1, we asked whether miR-212 could abrogate the autophagy inducing effects of SIRT1. LNCaP cells were cotransfected with EV or SIRT1 along with NC or miR-212 mimic under complete or serum free media conditions. Immunoblotting confirmed the induction of LC3B-II in both serum free and complete media upon SIRT1 transfection. SIRT1 transfection induced autophagy as shown by LC3B-II expression in both complete and in serum-deprived media (Figure 4). Further, co-transfection with miR-212 resulted in inhibition of SIRT1 levels and associated autophagy in both complete media and serum free conditions.

### miR-212 inhibits angiogenesis by modulating SIRT1 expression

**Figure 5:**
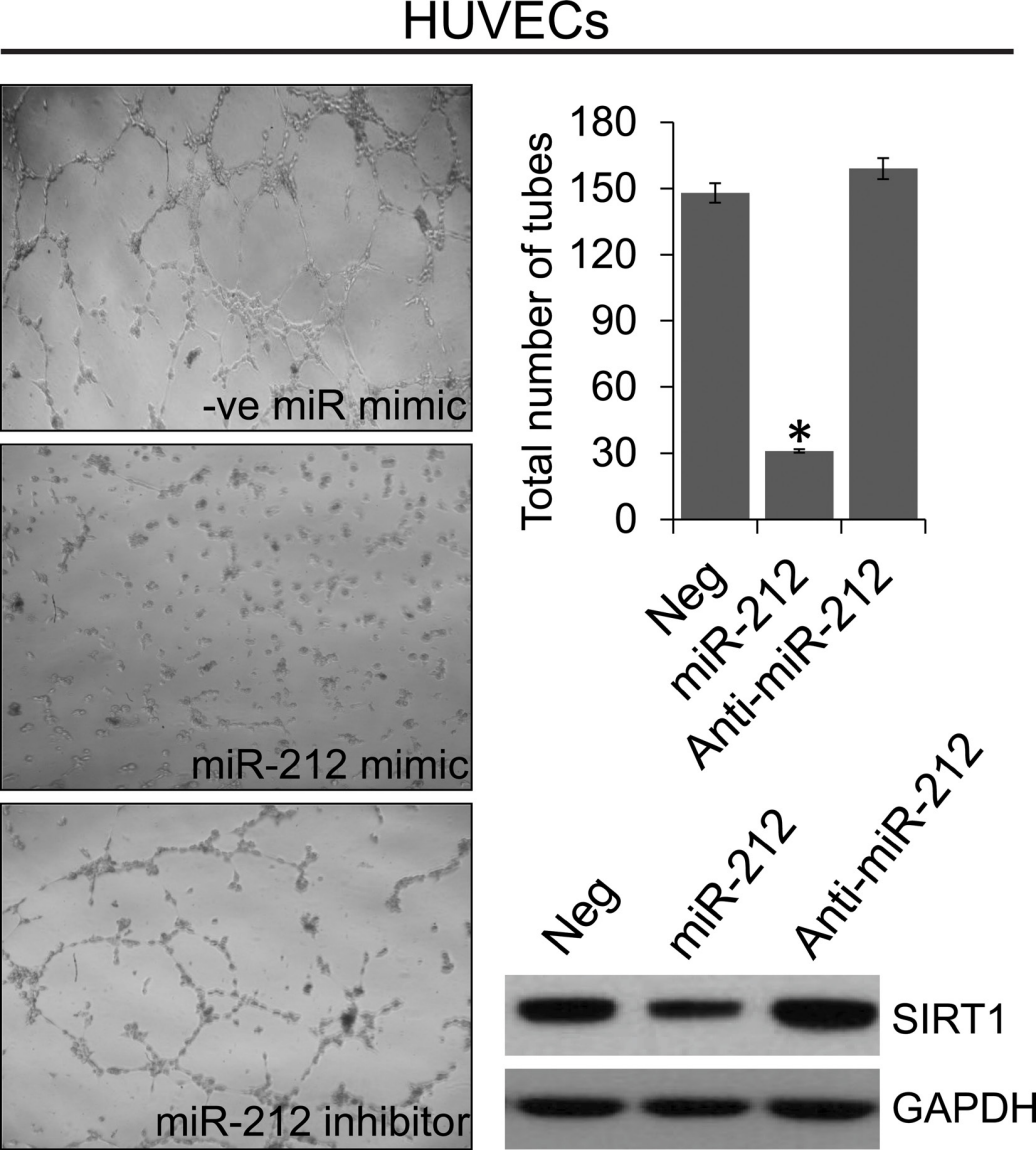
miR-212 inhibits angiogenesis HUVECs were transfected with NC, miR-212, or anti-miR-212 for 24 hours before being placed on the Matrigel. After 24 hours, phase-contrast images of the tube-like extensions were taken. Photomicrographs of representative Matrigel assay depicting the tube formation are shown along with quantification of tube counts. Inhibition of SIRT1 levels after transfection with miR-212 mimic was analyzed by immunoblotting. GAPDH was used as the loading control. *p < 0.005.

Vascular role of SIRT1 is well recognized [33, 34]. Since, SIRT1 is inhibited by miR-212, we assessed the effect of miR-212 on angiogenesis. To investigate whether miR-212 functions as an inhibitor of angiogenesis, miR-212 or NC or anti-miR-212 were transfected into human umbilical vein endothelial cells (HUVECs) cultured in EBM medium. Twenty-four hours after transfection, HUVECs were placed on Matrigel in the absence of angiogenic stimuli. miR-212 significantly inhibited tube formation and branching, in contrast to NC and miR-212 inhibitor (Figure 5). To understand if miR-212-induced repression of tube formation was associated with SIRT1 inhibition, immunoblot analysis was performed to determine the levels of SIRT1 expression. Our findings suggest that SIRT1 levels in HUVECs were significantly lowered by miR-212 mimic as compared with NC or anti-miR-212 and may be responsible for lower angiogenic differentiation.

### miR-212 induces cellular senescence by modulating SIRT1

**Figure 6:**
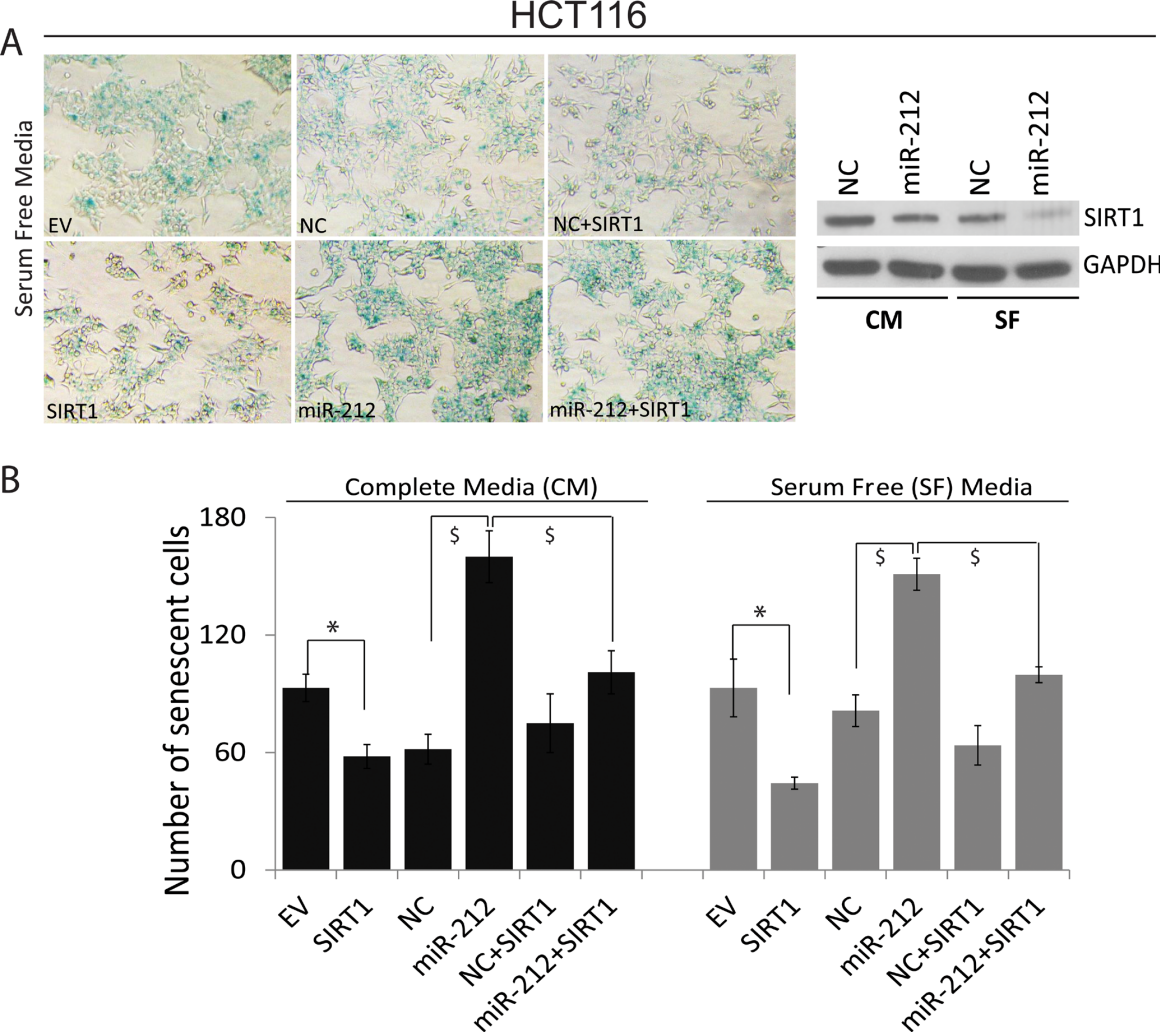
miR-212 induces cellular senescence HCT116 cells were transfected with NC or miR-212 mimic for 24h followed by changing to serum free and complete media for additional 24h. Cells were also transfected with empty vector and SIRT1 to rescue the effects of miR-212. After 48h of transfection, the cells were stained for senescence-associated (SA) β-galactosidase activity for evaluation of the senescent status at pH 5.8 at 37°C for 12h and images were taken at 100x magnification on Nikon Diaphot microscope. The β-gal staining-positive cells were counted and plotted. **A.** Left panel shows cells stained with SA-β-galactosidase in serum free media. Right panel shows inhibition of SIRT1 by miR-212 in both SF and CM. **B.** Quantification of SA-β-galactosidase positive cells in CM and SF media (*p < 0.005; $*p* < 0.001).

We have demonstrated that miR-212 inhibits SIRT1 expression. Decreased levels of SIRT1 are associated with premature senescence, an anti-proliferative response arresting cell cycle [35, 36]. We asked, whether miR-212 could modulate cellular senescence. Using HCT116 cells, we demonstrate that miR-212 inhibit endogenous SIRT1 levels in both serum free (SF) and complete media (CM) (Figure 6A, right panel). Further, in both SF and CM conditions, we show that transfection with miR-212 resulted in increased cellular senescence as shown by senescence associated β-galactosidase staining as compared to negative control mimic (Figure 6A and 6B). Pictures of SA-β-galactosidase positive cells are shown only for SF condition in 6A, left panel. Co-transfection with SIRT1 reduced the number of senescent cells in miR-212 transfected cells (Figure 6A, 6B) partially abrogating its senescence inducing affect.

### Clinical significance of miR-212 in prostate cancer: Expression analysis of miR-212 in tissue and serum from prostate cancer patients

**Figure 7:**
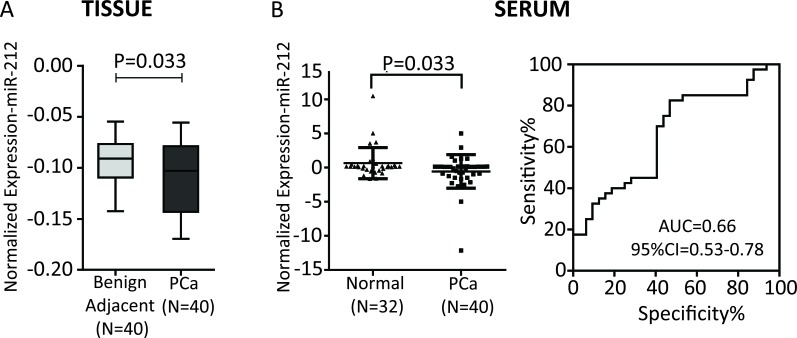
**A.** Expression of miR-212 in PCa tissues: Box plot representing tissue expression levels of miR-212 in PCa tissues and their paired benign adjacent tissues (*n*= 40 pairs of FFPE tissues). Significant decrease in miR-212 expression was observed in PCa tissues (*p*= 0.033) as compared to adjacent benign tissues. U6 snRNA was used as endogenous control. **B.** Expression of circulatory miR-212 in serum: Scatter plot representing the serum expression level of miR-212 in PCa patients (*n*= 40) and normal healthy controls (*n*= 32). Significant down regulation of miR-212 was observed in PCa patients (*P*= 0.033); miR-223 was used as control for serum studies as suggested by Kroh et al., (2010) [37]. Receiver operating characteristic (ROC) curve analysis of miR-212 was used to differentiate the PCa patients from healthy individuals. The area under the ROC curve (AUC) conveys its accuracy for differentiation of PCa patients and healthy subjects in terms of sensitivity and specificity.

To study the clinical significance of miR-212 function, we analyzed the expression of miR-212 in FFPE laser capture microdissected PCa tissues and benign adjacent tissue (BAT) in 40 patients as described earlier [18]. Expression level of miR-212 was compared between 40 PCa tissues and their corresponding BAT, which showed decreased expression of miR-212 in PCa tissue as compared with benign adjacent tissue (Figure 7A, *p*=0.033). Next we analyzed expression of circulatory miR-212 in serum from separate set of 32 Normal and 40 PCa patients as described [15]. The results showed that there was significantly low expression of miR-212 (Figure 7B-left, *p*= 0.033) in the serum of PCa patients as compared to that of normal individuals. We used miR-223 as internal control for serum studies as described earlier [15, 37]. Receiver operating characteristics (ROC) curves was constructed to explore the sensitivity and specificity of miR-212 as potential biomarkers to discriminate between cancer and disease free. The results suggests that miR-212 can fairly discriminate between the two groups (Figure 7B-right); AUC = 0.66 (95% CI-0.53 - 0.78). The data suggests that reduced expression of circulatory and tissue miR-212 expression may have functional role in PCa.

## DISCUSSION

In this study, we have demonstrated that miR-212 which is known to be downregulated in PCa, modulates autophagy, angiogenesis and senescence. During the initiation and progression of PCa, one or multiple molecular events disrupt the dynamic equilibrium between proliferation, apoptosis, autophagy and senescence [12, 31]. The observation that miR-212 modulates autophagy is highly significant and suggests an important role for loss of this miRNA in PCa. Autophagy plays an important role in cancer development and cancer cell survival modulating multiple tumor hallmarks [3]. Induction of autophagy has also been associated with development of resistance to chemotherapy and immunotherapy [9, 10]. Downregulation of miR-212 in PCa has also been demonstrated earlier [17]. In this study, we begin to seek the role of this miRNA in PCa by identifying its target. Our results show SIRT1 as miR-212 target. miR-212 and miR-132 are part of the same miR-132/212 cluster encoded by intron on chromosome 17. Both miR-212 and miR-132 share the same seed sequence and possibly targets. miR-132 is known to target SIRT1 [38, 39]. Our observations that miR-212 targets SIRT1, although new, supports earlier observations and indicates the functional role of repressed miR-212 in PCa by modulating SIRT1 related functions. Role of SIRT1 both as pro and anti-oncogenic molecule with autophagy modulating function has been reported [4, 5, 11, 12]. Autophagy initially serves as a protective process to prevent cancer initiation, however, after neoplastic transformation can promote tumor cell survival and maintenance. Our results suggest that miR-212 inhibits autophagy in PCa cells by inhibiting SIRT1. We confirmed the observations by inducing autophagy by serum starvation (Figure 3) and inducing SIRT1 (Figure 4). miR-212 has been shown earlier to modulate autophagy in cardiomyocytes [40]. Our observations are supporting and indicate the possible function for miR-212 in PCa. Loss of miR-212 in PCa may lead to induction of autophagy responsible for tumor promoting cell survival and aggressive disease [7, 41]. Autophagy is also induced in response to multiple cancer therapies limiting drug efficacy and has been attributed to development of drug resistance. It is plausible that restoring miR-212 in PCa may have therapeutic implications in PCa including CRPC. To further study anti-cancer potential of restoring miR-212, we studied the effects of miR-212 in modulating angiogenesis and senescence. Both angiogenesis and senescence play important roles in tumor progression and are known to be modulated by SIRT1 [11, 12, 31, 36]. Using HUVEC cells, we demonstrated that miR-212 negatively modulates angiogenesis (Figure 5) supporting earlier observations by Kumarswamy [38]. Nevertheless, the observations support cancer related therapeutic implications of miR-212. The role of cellular senescence in inhibiting tumorigenesis is well studied [42–45]. Also, SIRT1 prevents cellular senescence possibly contributing to its pro-survival functions [36, 46–48]. Since, miR-212 inhibits SIRT1; we asked whether miR-212 could modulate cellular senescence. We demonstrated that miR-212 induces cellular senescence in HCT116 colon cancer cells supporting the anti-cancer role of miR-212 (Figure 6). Our observations that miR-212 is negatively modulated both in PCa tissues and serum from PCa patients is corroborative and supports the important role miR-212 in PCa. Systemic negative modulation of miR-212 in PCa patients may lead to modulation of autophagy, angiogenesis and cellular senescence. The observations in this study clearly suggest therapeutic potential of miR-212 and support the development of miR-212 based therapies.

## MATERIALS AND METHODS

### Cell culture and antibodies

The prostate cancer cell lines LNCaP and PC3 and HCT116 colon cancer cells were obtained from Georgetown University (GU) Lombardi Comprehensive Cancer Center (LCCC) cell culture repository and cultured in DMEM (Invitrogen, Carlsbad, CA) medium supplemented with 10% fetal bovine serum (FBS), 2mM-glutamine, 25ug/ml of gentamicin (Invitrogen) and incubated at 37^◦^C with 5% CO_2_. Human Umbilical Vein Endothelial Cells (HUVECs) were purchased from Lonza (Allendale, NJ) and cultured as described [49]. The following primary antibodies: SIRT1, LC3B-I/II, and GAPDH antibodies were purchased from (Cell Signaling Technology Inc., Danvers, MA). Secondary antibodies conjugated with horseradish peroxidase were from Jackson ImmunoResearch (West Grove, PA).

### MiRNA mimics, inhibitors, plasmids and transfections

MicroRNA miR-212 mimics with sequence UAACAGUCUCCAGUCACGGCC (AM17100) and negative control (NC) (AM17110) were purchased from Invitrogen. Anti-miR-212 (miRCURY LNA microRNA 212 inhibitor: 410138-04) was from Exiqon (Woburn, MA) and FLAG-tagged SIRT1 expression vector (EX-U1443-M11) from Genecopoeia (Rockville, MD). For transfections, the cells at 50-60% confluence were transfected with miR-212 mimic or inhibitor (50 nM) and NC (50 nM) by using lipofectamine 2000 (Invitrogen).

### Western blot analysis

Immunoblotting analysis was performed following standard procedures. The cells were lysed with cell lysis buffer (Cell Signaling, Danvers, MA) containing protease inhibitor mixture (Roche, Indianapolis, IN). Protein estimations were performed using Bio-Rad protein assay (Bio-Rad, Hercules, CA). The proteins (30 μg) were separated on 4-12% Bis-Tris gel (Invitrogen) and transferred on to the PVDF (Immobilon-P, Millipore, Billerica, MA). Membranes were blocked in 1x blocking buffer (Sigma-Aldrich, St. Louis, MO) and incubated in primary antibody overnight at 4^◦^C. After washing, the membranes were incubated in secondary antibody for 1 hour at room temperature and visualized with chemiluminescence detection (Signagen, Rockville, MD).

### 3′UTR luciferase assay

HEK293 cells were seeded in a 12 well plate and co-transfected with 200 ng of SIRT1-3′UTR-Luciferase reporter vector and 50 nM of miR-212 or NC miRNA by using Lipofectamine 2000 according to the manufacturer's instructions. Transfected cells were lysed after 24 hours of transfection and luciferase activity was assayed using light switch assay system (Switchgear Genomics, Carlsbad, CA) following manufacturer's instructions.

### Angiogenesis assay

Angiogenesis was assessed by tube formation assay in HUVEC cells. For the tube formation assay, HUVECs were transfected with NC mimic, miR-212 mimic or anti-miR-212 and SIRT1 expression plasmid. After 24 hours, 50 μL of Matrigel (ECM 625; Millipore, Billerica, MA) was tiled on the bottom of 96 well plates at 4°C and left at 37°C for gel formation. HUVECs (5×10^5^) were then seeded on the Matrigel and incubated in endothelial basal medium (EBM; Lonza, Allendale, NJ) at 37°C with 5% CO_2_. After 24 hours of incubation, the tube-like structures were visualized and bright-field images were captured at 400x magnification using the Nikon Diaphot microscope. Tube formation was assessed by counting the number of tube branches per viewing field.

### Senescence associated β-galactosidase (SA-β-gal) staining

Senescence was assessed by SA-β-gal staining of HCT116 cells transfected with indicated mimics or expression vectors. After 24hrs post transfection the cells were cultured in complete and serum free media followed by staining using senescence β-galactosidase staining Kit (Cell Signaling, Danvers, MA) following manufacturer's instructions. Briefly, the cells were rinsed with PBS and fixed (2% formaldehyde, 0.2% glutaraldehyde in PBS) for 15 min at room temperature and washed twice with PBS. The cells were incubated with fresh β-galactosidase staining solution at pH 5.8 and incubated at 37°C for overnight. SA-β-galactosidase positive cells were detected by inverted bright field microscopy (Nikon Diaphot) at 100x magnification.

### Serum samples

Serum samples from 40 PCa patients (16 Caucasian Americans (CA) and 24 African Americans (AA)) were obtained from GU-IRB-approved and consented Georgetown University Hospital Cyberknife Prostate Cancer Program from 2009 to 2012. Age matched serum samples from 32 healthy individuals (20CA and 12AA) were obtained from Innovative Research (Novi, Michigan) and Georgetown University Hospital. All the patients serum samples obtained were de-identified to protect patient confidentiality.

### Tissue samples and laser capture microdissection

Archived forty formalin-fixed, paraffin-embedded (FFPE) tissue specimen blocks from radical prostatectomy consisting 15 Caucasian American (CA) and 25 African American (AA) were obtained from GU/LCCC Histopathology & Tissue Shared Resource (http://lombardi.georgetown.edu/research/sharedresources/htsr/) and processed for LCM and RNA isolation as described earlier [18].

### RNA extraction and qRT-PCR

RNA extraction from microdissected cells and serum samples was performed essentially as described earlier [15, 16, 18]. RNA was quantified using NanoDrop ND-1000 Spectrophotometer (Thermo Scientific, Waltham, MA). The miR-212 expression levels were measured as described earlier [15, 18] by qRT-PCR using inventoried TaqMan^®^ miRNA assay (Applied Biosystems, Grand Island, NY) following manufacturer's recommendations, on 7300 Real-Time PCR System (Applied Biosystems). miR-223 was used as control for serum studies.

### Statistical analysis

All qRT-PCR experiments were conducted according to the MIQE (minimum information for publication of quantitative realtime PCR experiments) as described [15]. The nonparametric Student's T-test was used for comparing two groups. For paired PCa and benign adjacent samples, Wilcoxon signed-rank test was used. Receiver operating characteristic (ROC) curves were constructed and area under curve (AUC) was estimated to study the feasibility of using the particular miRNA to discriminate PCa patients from healthy controls. Logistic regression was used to construct ROC curves using miRNA expression levels. Data are presented as means ± SE. P value of p ≤ 0.05 was considered statistically significant. All the statistical analyses were performed using GraphPad Prism (La Jolla, CA).

## Abbreviations

PCa: Prostate cancer
CRPC: Castrate resistant prostate cancer
SIRT: Sirtuin
NAD: Nicotinamide adenine dinucleotide
AR: Androgen Receptor
Rb: Retinoblastoma
FOXO1: Forkhead box O1
Atg5: Autophagy protein 5
GAPDH: Glyceraldehyde 3-phosphate dehydrogenase
NC: Negative control
3′UTR: 3′ untranslated region
SFM: Serum free media
CM: Complete media
HUVECs: Human umbilical vein endothelial cells
FFPE: Formalin-fixed, paraffin-embedded
BAT: Benign adjacent tissue
ROC: Receiver operating characteristic
SA-β-gal: Senescence associated β-galactosidase
